# Evaluating the Alterations Induced by Virtual Reality in Cerebral Small-World Networks Using Graph Theory Analysis with Electroencephalography

**DOI:** 10.3390/brainsci12121630

**Published:** 2022-11-28

**Authors:** Shan Yang, Hyeon-Sik Hwang, Bao-Hua Zhu, Jian Chen, Ganbold Enkhzaya, Zhi-Ji Wang, Eun-Seong Kim, Nam-Young Kim

**Affiliations:** 1 RFIC Center, Department of Electronic Engineering, Kwangwoon University, Seoul 01897, Republic of Korea; 2 NDAC Center, Kwangwoon University, Seoul 01897, Republic of Korea; 3 Department of Pediatrics, Severance Children’s Hospital, Yonsei University, Seoul 03722, Republic of Korea; 4 WAVEPIA Co., Ltd., 557, Dongtangiheung-ro, Hwaseong-si 18469, Republic of Korea

**Keywords:** electroencephalography, virtual reality, graph theory, small-world networks, two-streams hypothesis, betweenness centrality

## Abstract

Virtual reality (VR), a rapidly evolving technology that simulates three-dimensional virtual environments for users, has been proven to activate brain functions. However, the continuous alteration pattern of the functional small-world network in response to comprehensive three-dimensional stimulation rather than realistic two-dimensional media stimuli requires further exploration. Here, we aimed to validate the effect of VR on the pathways and network parameters of a small-world organization and interpret its mechanism of action. Fourteen healthy volunteers were selected to complete missions in an immersive VR game. The changes in the functional network in six different frequency categories were analyzed using graph theory with electroencephalography data measured during the pre-, VR, and post-VR stages. The mutual information matrix revealed that interactions between the frontal and posterior areas and those within the frontal and occipital lobes were strengthened. Subsequently, the betweenness centrality (BC) analysis indicated more robust and extensive pathways among hubs. Furthermore, a specific lateralized channel (O1 or O2) increment in the BC was observed. Moreover, the network parameters improved simultaneously in local segregation, global segregation, and global integration. The overall topological improvements of small-world organizations were in high-frequency bands and exhibited some degree of sustainability.

## 1. Introduction

Virtual reality (VR) simulates an artificial sensory world where users can interact with various virtual items and environments, emerging as an integrated stimulus, especially in the cortical system [1,2]. This flexible, immersive, and user-friendly interaction technology can improve cognitive and memory functions [3,4]. This functional improvement is achieved by activating neuroplasticity, the process by which the cortex encodes experiences and learns new behaviors in response to environmental changes [5,6]. Identifying the correlation between VR intervention and complex dynamic neurophysiological events has received considerable attention [7].

Human cognition is a spontaneous mental process supported by a functional network, which is a dynamic statistical dependency based on an anatomical substrate [8,9]. Graph theory has been applied across disciplines in network neuroscience to represent intricate functional networks [10]. It is a mathematical description of a set of nodes and their edges to evaluate the topological properties of functional connectivity, such as small-worldness, modularity, and centrality [11,12]. Thereinto, small-world architectures are networks in which a concrete node can always reach others in a few steps although most are indirectly connected, reconciling the opposing demands of integration and segregation [13,14]. Functional connections among cortical regions are consistent with a small-world network, whose segregation and integration can satisfy resource cost minimization and communication efficiency maximization among modules, respectively [15]. Evidence suggests that the small-worldness of functional connectivity undergoes successive topological changes with various cognitive loads. This process is based on neuroplasticity, the ability to rapidly grow and reorganize distributed neural networks ranging from new pathways among individual neurons to routine adjustments [16,17]. These alterations may provide novel insights into the neurophysiological mechanisms induced by VR.

Electroencephalography (EEG), a technique used to assess the complex cortical connectivity pattern, can measure the brain’s electrical signals non-invasively with good time resolution [18]. Recently, researchers have shown increased interest in identifying VR-induced neuroplasticity by measuring EEG data. Luca et al. calculated the interdependence among regions of interest and observed a significant change in several frontoparietal connections after VR [19]. Filho et al. discovered significant changes in functional connectivity following VR intervention and further quantitatively analyzed metrics, such as degree, clustering coefficiency (CC), betweenness centrality (BC), and characteristic path length (CPL). However, they observed no significant variation in the last variable [20]. These studies have observed that the amount and intensity of functional connections are dynamic; however, they only applied graph theory to EEG recordings of pre- and post-VR resting states. Regarding the EEG data measured during VR, published works employ event-related potential (ERP) analysis rather than graph theory to explore the discharge response of a specific cortex to VR stimuli, which are repetitive task-oriented rather than scenario-oriented [21,22,23]. Moreover, studies have focused on VR-induced neurophysiological events in regions of interest rather than on global functional network characteristics, especially the small-world architecture. Accordingly, much uncertainty remains about the functional links during VR intervention and the continuous alterations in small-worldness before, during, and after VR.

In this study, we assumed that VR intervention induces neuroplasticity, thereby altering functional connectivity and improving small-worldness. Therefore, based on graph theory, we aimed to identify and interpret VR-mobilized neuroplasticity by assessing topological properties before, during, and after VR. To model brain dynamics, graphical representations and multiple network metrics were obtained to cover different aspects of functional connectivity and characterize successional alterations in small-world networks. Our results reflect that VR can result in a widely distributed and highly hierarchical small-world organization in high-frequency bands.

## 2. Materials and Methods

**Figure 1:**
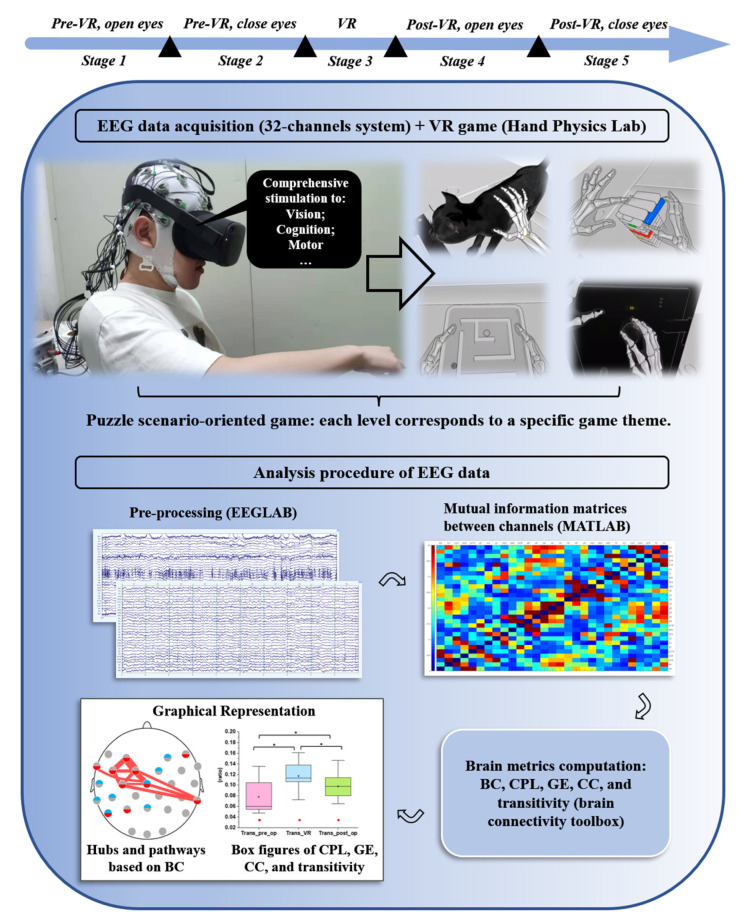
Depiction of this experiment: the sequence of the participants’ different stages and the procedures of acquiring and analyzing EEG data.

The study procedure was conducted following the Declaration of Helsinki and was approved by the Institutional Review Board (IRB) of Kwangwoon University, Seoul, South Korea (IRB No. 7001546-20210928-HR(BM)-009-01). Figure 1 depicts the whole experimental paradigm.

### 2.1. Participants

We placed advertisements at Kwangwoon University to recruit healthy volunteers for this experiment. Participants were asked to complete a questionnaire and test levels 1–5 of a VR game named the Hand Physics Lab (https://www.holonautic.com/hand-physics-lab (accessed on 28 Novemeber 2022)). In this game, users can fully control their virtual hands and fingers to play with different virtual objects, such as toy bricks, finger paints, and puzzle pieces, to complete unique missions for each level. Fourteen participants (age: 27 ± 6; eight males, six females) were selected based on the following inclusion criteria:

1. Healthy participants who were proven to possess normal vision, hearing, and cognition by passing game levels 1–5
2. Age between 20 to 35 years
3. Right-handedness
4. No drinking, coffee consumption, or smoking in the past 24 h
5. No experience with VR or other three-dimensional games
6. No addiction to two-dimensional games

The experimental design and each test step were explained to the participants before the experiment. Participants were informed that they could stop the experiment at any time if they felt uncomfortable. Subsequently, they signed an informed written consent form provided by the IRB.

No subject reported significant symptoms of discomfort and asked to stop the experiment. But four participants with poor EEG data quality were excluded from our analysis and discussion, because of a high degree of artifact contamination in all EEG channels, caused by extensive head movements and severe friction between the VR headset and EEG electrodes. Hence our study finally included ten participants (age: 26 ± 6; seven males, three females).

### 2.2. EEG Recordings

EEG activity was recorded using a 32-channel actiCAP active wet electrode system (Brain Products, Munich, Germany) and an actiCAP electrode cap (Brain Products, Munich, Germany). High-quality Ag/AgCl (sinter) electrodes were placed following the international 10-10 system. Two electrodes with poor signal quality (Fp1 and Fp2) were excluded because the VR eyeshade covered them. An electrode placed at Fz was used as the reference electrode. Subsequently, EEG data were recorded at a sampling frequency of 500 Hz using 29 electrodes (F7, F3, F4, F9, FT9, FC5, FC1, FC2, FC6, FT10, T7, C3, Cz, C4, T8, TP9, CP5, CP1, CP2, CP6, TP10, P7, P3, Pz, P4, P8, O1, Oz, and O2). Each electrode was integrated with color light-emitting diodes (red, yellow, and green) to indicate the resistance threshold. The resistance of each electrode was lower than 2 kΩ by the indication of electrode lamps before recording.

Regarding the experimental procedure, the participants were asked to play the Hand Physics Lab using an Oculus VR headset (Oculus quest headset), which provided an immersive and interactive environment. During the game, players watched virtual environments, considered how to pass game levels, and freely used their hands and fingers to interact physically with various virtual objects. It stimulates the participants’ visual, cognitive, and motion neural regions. To prevent fatigue after a long time, the participants were only required to go through the levels 6–25, from easy to hard, without any guidance.

The EEG data were recorded in five stages.

Stage one: Open-eyes without a task for 5 min
Stage two: Close-eyes without a task for 5 min
Stage three: Play the VR game for approximately 20 min
Stage four: Open-eyes without a task for 5 min
Stage five: Close-eyes without a task for 5 min

After each stage, the participants rested for 5 min before proceeding to the next stage. Throughout the procedure, participants sat comfortably in a noise-free recording room with constant room temperature and humidity to avoid physical environment disruption.

The stage one with task-free and open-eyes condition was recorded to serve as a baseline of stage three with VR task and open-eyes. Afterward, we recorded stage four under the same condition as stage one to assess VR-induced neuroplasticity that could even be preserved after VR stimuli. Both stage two and stage five with task-free and closed-eyes conditions were the typical resting-states, which could capture the endogenous neuronal synchronization in the absence of tasks [24]. We compared EEG data in this resting-state pre- and post-VR to further explore the VR-elicited aftereffects on intrinsic neural activity [25].

### 2.3. EEG Processing

#### 2.3.1. EEGLAB (v2021.0) [26]

Raw EEG data and electrode location files were imported into the EEGLAB software (v2021.0). We used a 0.53 Hz high-pass filter and a 59–61 Hz notch filter to remove the direct current offset and power line noise, respectively.

Ocular artifacts were eliminated using the blind source separation algorithm in the automatic artifact removal toolbox, which can determine the origin of the multi-sensor-generating EEG signals and remove the ocular-artifact components to reconstruct the data [27]. Additionally, the multivariate EEG signals were decomposed into statistically independent components using the infomax independent component analysis (ICA) algorithm. The artifact components caused by heartbeats, nods, and movements of eyeglasses were identified by the energy topography and frequency spectrum and then removed to reduce residual noises [28].

#### 2.3.2. MATLAB (vR2021a) and the Brain Connectivity Toolbox (v2019_03_03) [29]

The whole preprocessed continuous EEG data were consecutively segmented into one-second epochs, which were subsequently exported in ‘txt’ format. Only 50 clean epochs were chosen and loaded into MATLAB (vR2021a) for each recording period because the ICA and automatic artifact removal algorithms could not remove muscle artifacts caused by frequent arm and hand movements in games. The epochs were divided into six commonly used frequency bands using a bandpass filter: delta (1–4 Hz), theta (4–8 Hz), alpha (8–13 Hz), beta (13–32 Hz), low gamma (32–50 Hz), and high gamma (50–80 Hz). The dependence between functional network channels in each stage was analyzed based on mutual information (MI). We calculated the correlation between MI channels for each epoch by a bin-based method with the Freedman–Diaconis rule, and then obtained the stage MI matrix by averaging MI over all 50 epochs. For every stage, the elements of weighted connection matrix were normalized and rescaled to the absolute value ranging from zero to one by the ‘weight conversion’ function in the brain connectivity toolbox. Alternatively, if the activities from both channels were independent of each other, the value of the MI between them would be quite low. In contrast, this value would be high if there is robust communication [30]. All the MI matrices were visually represented by MATLAB software, with different shades of blue and red squares corresponding to the magnitude of negative and positive values in the matrices, respectively. Subsequently, five network metrics were calculated in the brain connectivity toolbox: BC, CPL, CC, global efficiency (GE), and transitivity. Finally, box plots were used in OriginPro 2018 (OriginLab Corporation, Northampton, MA, USA) to express alterations in the network parameters.

### 2.4. Statistical Analyses (IBM SPSS Statistics Version 26)

Analysis of variance (ANOVA) is a statistical method for categorizing observed aggregate variability into systematic and random factors. The former statistically influences the given dataset, whereas the latter does not. When only one within-subject factor exists, one-way repeated-measures ANOVA can be used to compare whether the difference between the dependent variables is significant or null. The null hypothesis is rejected if the *p*-value is less than 0.05. In this study, all metrics in the six different frequency bands were processed using a one-way ANOVA test, and statistical significance *p* was set at 0.05. Subsequently, the post-hoc *t*-test was used to statistically evaluate the difference between each sub-paired group (pre- and during VR, during and post-VR, and pre- and post-VR), and the statistical significance p for each group was set at 0.05/3 or 0.016 with Bonferroni correction. Finally, the significant differences in network parameters between stages 5 and 2 were evaluated using a paired-samples t-test. The dependent variables were network metrics, and the independent variable was the stage. Additionally, the Mann–Whitney U test was used to assess gender differences in global metrics, including CPL, GE, and transitivity for all stages 1–5. The indicators significantly differed between males and females if the *p*-value was <0.05.

### 2.5. Network Measures and Small-World Network

*BC* can capture the numbers of a node to all shortest paths through it and measure a region’s effectiveness on information flow across all functional networks [31]. Therefore, hubs are nodes with considerable *BC* values [32].

(1) $$BC\left(i\right)=\frac{2}{\left(N-1)(N-2\right)}\sum_{h\neq j\neq i}\frac{n_{hj}(i)}{n_{hj}}$$

where *N* is the number of nodes, *n_hj_(i)* is the number of shortest paths between nodes *h* and *j* passing through node *i*, and *n_hj_* is the number of shortest paths between the two nodes.

CPL is the average of the shortest paths between all vertex pairs. CPL can directly measure the average path length in the paired nodes and is inversely proportional to the degree of integration. However, in the disconnected network, the path length between disconnected node pairs is defined to be infinite, which would lead to a quite large CPL value and further influence the accuracy of measured functional integration [33]:

(2) $$L=\frac{1}{N}\sum_{i}l_{i}$$

where *l_i_* is the average of the shortest paths of node *i* to other nodes.

*GE* is a relative measure based on the inverse of CPL. In the disconnected network, the efficiency of disconnected node pairs is inversely defined as zero value and does not influence the total efficiency, which can lead to a more valid measurement of the functional integration [33]:

(3) $$GE=\frac{1}{N(N-1)}\sum_{i\neq j}\frac{1}{l_{ij}}$$

where *l_ij_* is the shortest path between nodes *i* and *j*.

*CC* is calculated using the fraction of triangles around a node to evaluate local segregation, whereas transitivity is a normalized variant of *CC* that reflects global segregation [34]:

(4) $$CC\left(i\right)=\frac{2t_{i}}{k_{i}(k_{i}-1)}$$

(5) $$t=\frac{\sum_{i}t_{i}}{m}$$

where *k_i_* is the number of edges between node *i* and other nodes, *t_i_* is the number of triangles around node *i*, and m is the number of connected triples of the nodes in the network.

The integration of the whole functional network can be evaluated using CPL and *GE* values. In contrast, the local segregation of each node and global segregation can be demonstrated using *CC* and transitivity, respectively.

**Figure 2:**
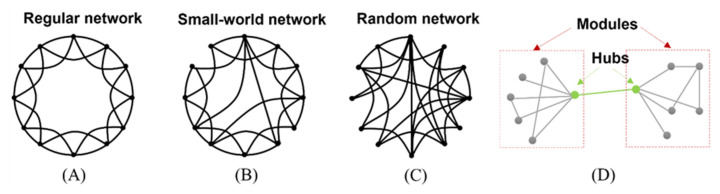
Different kinds of networks and the graphical introduction of nodes and links: (**A**) Regular network; (**B**) Small-world network; (**C**) Random network; (**D**) Graphical introduction of hubs and modules.

Networks can be divided into regular, small-world, and random networks (Figure 2A–C). The regular network, a geometrically symmetric model with several clusters, always has an unusually long path length, resulting in a high CPL value. Conversely, the random network lacks geometric symmetry and has few clusters, resulting in a short CPL. The small-world network combines characteristics of the former two networks with short CPL and several clusters that are highly segregated and integrated simultaneously. Thus, small-world networks have significant modularity, which is the tendency of a group of nodes in the network to interconnect densely but connect sparsely with external nodes via hubs (Figure 2D). Functional networks of the cortex are consistent with small-world architectures, which drives the brain system’s low energy and wiring costs through a dynamic equilibrium between information segregation and integration [35].

## 3. Results

In our study, VR strengthened local mutual interactions, activated more robust and extensive pathways among functional modules, and improved global integration, global segregation, and local segregation simultaneously. All alterations were concentrated in the high-frequency (beta, low gamma, and high gamma) bands because responses to cognitive loads and visual stimuli were dominantly found in these bands [20,31,36]. Therefore, this study focused on high-frequency bands.

### 3.1. The MI Difference between VR and Pre-VR in Open Eyes, and Post-VR and Pre-VR in Open Eyes

**Figure 3:**
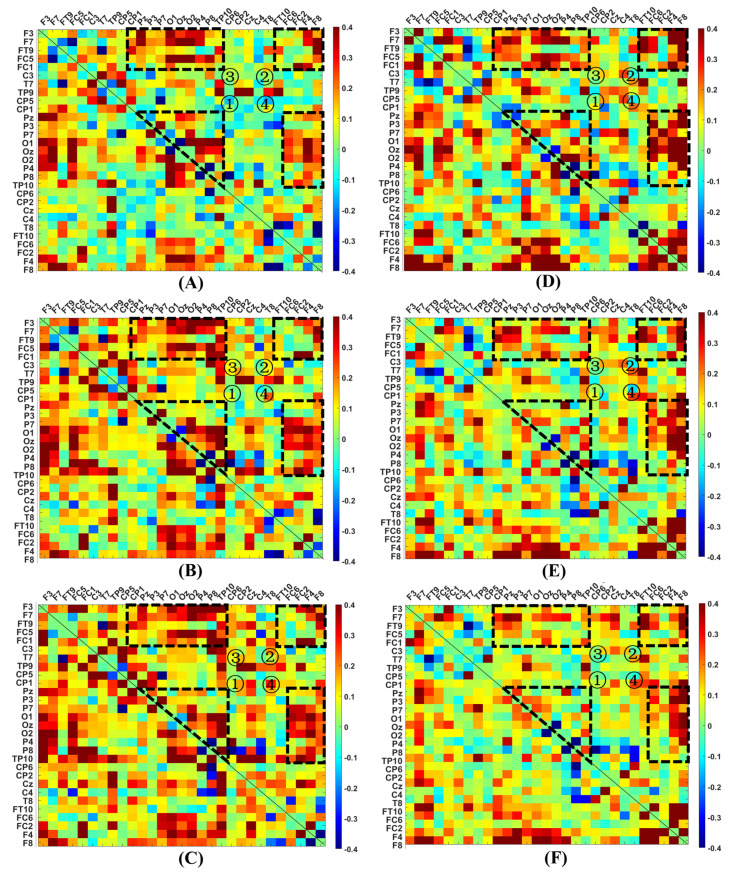
MI difference between stages three and one, and between stages four and one at different frequency bands: (**A**) MI difference between stages three and one in the beta band; (**B**) MI difference between stages three and one in the low gamma band; (**C**) MI difference between stages three and one in the high gamma band; (**D**) MI difference between stages four and one in the beta band; (**E**) MI difference between stages four and one in the low gamma band; (**F**) MI difference between stages four and one in the high gamma band. Four blocks of improvement were marked as ①–④.

In our research, an MI matrix with a size of 29 × 29, corresponding to 29 EEG recording electrodes, was used for each stage. The difference in MI between stages three and one in the beta, low gamma, and high gamma bands demonstrates the continuous variations in interactions between channels induced by VR (Figure 3A–C). Figure 3D–F depict the MI difference between stages four and one in the beta, low gamma, and high gamma bands. The corresponding MI matrix values in Figure 3 were provided in the supplementary files. Furthermore, supplementary Figures S1–S2 show the case of the alpha band. Color scales of [−0.4, 0.4] are set for the normalized MI matrix to attenuate the influence of individual extreme values because almost all MI matrix values are in the range of [−0.55, 0.55], except for individual values close and equal to one. Supplementary Figure S3 depicts the distribution of the top 30% correlations in Figure 3A,D. There are several regions larger than 24 grids, where more than half of the correlations correspond to these top 30% values. We combined blocks ①–④ to label them in the half plot above the diagonal at all frequency bands in Figure 3 because of the MI matrix symmetry. Considering these blocks, the electrode nodes in block ① (Pz, P3, P7, O1, Oz, O2, P4, P8, and TP10) and block ② (FT10, FC6, FC2, F4, and F8) belonged to the posterior and frontal areas, respectively. The correlations between two groups of electrodes in block ③ (group 1: F3, F7, FT9, FC5, FC1; group 2: CP1, Pz, P3, P7, O1, Oz, O2, P4, P8. TP10) and in block ④ (group 1: FC6, FC2, F4, F8; group 2: Pz, P3, P7, O1, Oz, O2, P4, P8, TP10) corresponded to the interaction between the left frontal and posterior cortices, and the interaction between the right frontal and posterior cortices, respectively. We observed significantly increased interactions within the posterior nodes and frontal area, and between the occipital and frontal areas in both contrasting groups. Moreover, the interaction in the posterior-to-anterior pattern and intra-frontal nodes was more marked than in the posterior sub-network in contrasting stages four and one. Additionally, the MI values between stages five and two in the alpha, beta, low gamma, and high gamma groups were compared (Supplementary Figure S4).

### 3.2. Alterations in BC Caused by Immersive VR Intervention

**Figure 4:**
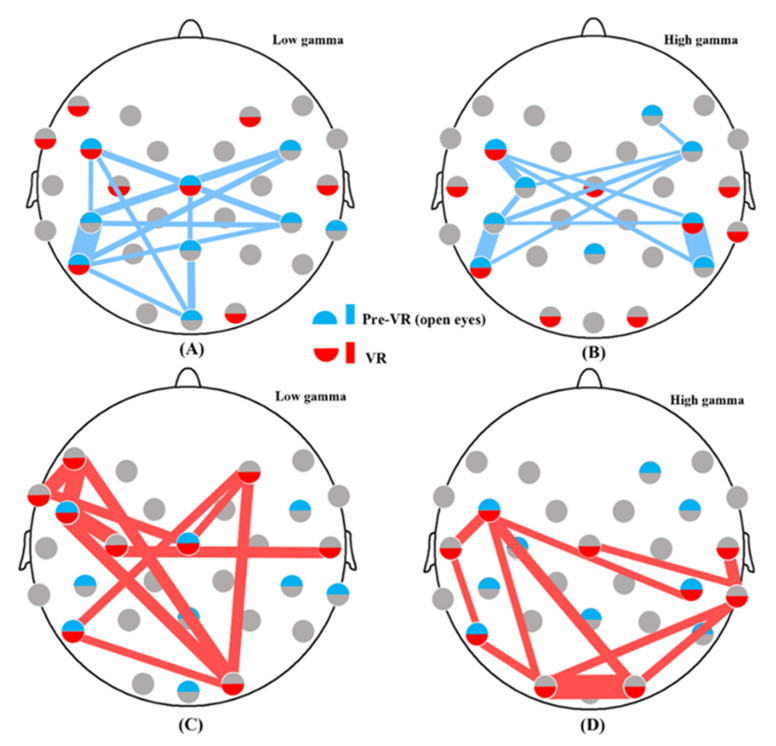
Variations of the top 30% connections among hubs in stages one and three at different frequency bands: (**A**) The top 30% of hub connections in stage one in the low gamma band; (**B**) The top 30% of hub connections in stage one in the high gamma band; (**C**) Top 30% of hub connections in stage three in the low gamma band; (**D**) The top 30% of hub connections in stage three in the high gamma band. The connection width is proportional to the MI value.

Considering functional network analysis, hubs are nodes with the top 30% of BC values [32]. In our study, there should be 29 × 0.3 ≈ 9 hubs, and the MI among these hubs in stages one and three are compared (Supplementary Figure S5). We discovered that mutual communication between hubs was strengthened during the VR intervention in the low and high gamma bands; however, it was not strengthened in the beta band. To comprehend the interactions between hubs more intuitively, the top 30% of the MI values were used to evaluate the most forceful pathways in a specific network. The three-dimensional VR game can activate more even, robust, and widespread connectivity in the low and high gamma bands (Figure 4). Considering the low gamma band, the functional links within the frontal lobe and between the frontal and occipital lobes were more intense and complex in the VR intervention. Moreover, interactions in the high gamma band were concentrated in the central lobe in Stage one. In contrast, a series of information flows between the occipital, temporal, and ventrolateral prefrontal areas emerged during the VR intervention.

**Figure 5:**
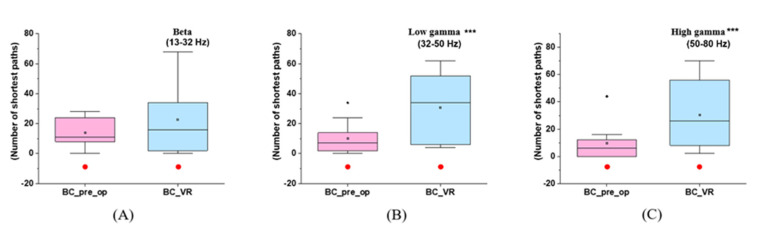
BC variations of a lateralized occipital channel (O1 or O2) in stages one and three at different frequency bands: (**A**) BC in the beta band (*p* = 0.33); (**B**) BC in the low gamma band (*p* = 0.01); (**C**) BC in the high gamma band (*p* = 0.008). *** indicates that the difference in the diagram is significant (*p* < 0.05) in the paired–samples *t*-test. The solid red circle indicates no gender difference for this parameter in this stage.

The frontal, parietal, temporal, and occipital lobes correspond to the cognitive, somatic, memory, and visual functions, respectively. Moreover, the longitudinal fissure divides the brain into left and right hemispheres, resulting in functional lateralization. More specifically, the right hemisphere dominates the visual, tactile, and motor functions on the left side of the body, and vice versa [37,38]. Every human has a dominant eye that provides slightly more inputs to the visual cortex and relays location information more accurately [39]. In this study, because the immersive VR game served as an intense visual stimulus, the BC values of the single-channel (O1 or O2) in the high-frequency bands were assumed to always increase, which could be evaluated by the difference in BC between stages one and three. Considering our experiment, as illustrated in Figure 5 and Supplementary Table S1, there is always a significant promotion of BC values in a specific channel (O1 or O2) in both the low (*p* = 0.01) and high (*p* = 0.008) gamma bands; nonetheless, this is not the case with the beta band.

### 3.3. Effects of Immersive VR Interventions on Functional Integration and Segregation of the Brain Network

**Figure 6:**
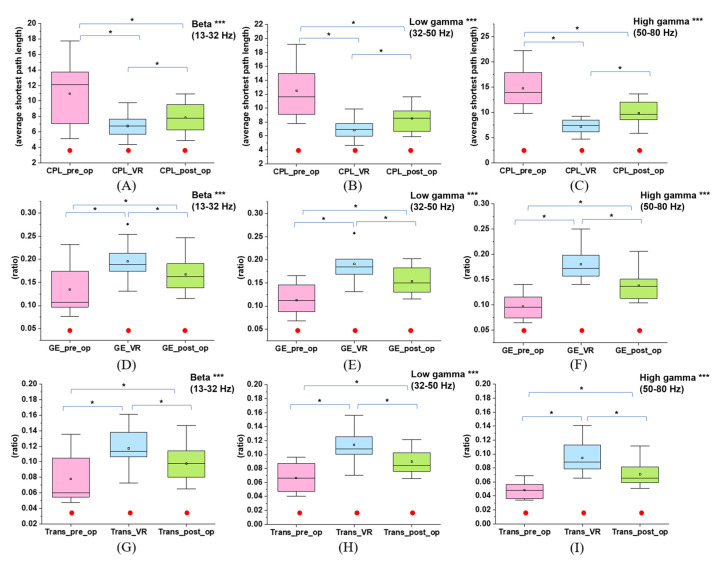
CPL, GE, and transitivity variations in stages one, three, and four at different frequency bands: (**A**) CPL in the beta band (*p* < 0.001); (**B**) CPL in the low gamma band (*p* < 0.001); (**C**) CPL in the high gamma band (p < 0.001); (**D**) GE in the beta band (*p* < 0.001); (**E**) GE in the low gamma band (*p* < 0.001); (**F**) GE in the high gamma band (*p* < 0.001); (**G**) Transitivity in the beta band (*p* < 0.001); (**H**) Transitivity in the low gamma band (*p* < 0.001); (**I**) Transitivity in the high gamma band (*p* < 0.001). *** indicates that the difference in the diagram is significant (*p* < 0.05) in the one-way repeated measures ANOVA, and * indicates that the difference between the sub–paired samples is significant (*p* < 0.016) in the post–hoc *t*-test. The solid red circle indicates no gender difference for this parameter in this stage.

We compared the three global indicators (CPL, GE, and transitivity) between stages one, three, and four to explore the effects of VR in the high-frequency bands (Figure 6 and Supplementary Table S2). VR could cause changes in network metrics, which was statistically verified by the one-way repeated measures ANOVA: in the beta band, CPL (*p* < 0.001, η^2^ = 0.614), GE (*p* < 0.001, η^2^ = 0.763), and transitivity (*p* < 0.001, η^2^ = 0.780); in the low gamma band, CPL (*p* < 0.001, η^2^ = 0.789), GE (*p* < 0.001, η^2^ = 0.866), and transitivity (*p* < 0.001, η^2^ = 0.886); in the high gamma band, CPL (*p* < 0.001, η^2^ = 0.791), GE (*p* < 0.001, η^2^ = 0.865), and transitivity (*p* < 0.001, η^2^ = 0.829). Furthermore, the p-values of the post-hoc *t*-test were all less than 0.016 for each sub-paired group that evaluated the VR-induced positive changes: CPL_stage1_ > CPL_stage4_ > CPL_stage3_, GE_stage3_ > GE_stage4_ > GE_stage1_, and transitivity_stage3_ > transitivity_stage4_ > transitivity_stage1_. In addition, CC_stage3_ > CC_stage4_ > CC_stage1_ were acquired in most channels with significance in the high-frequency bands (Supplementary Table S3).

**Figure 7:**
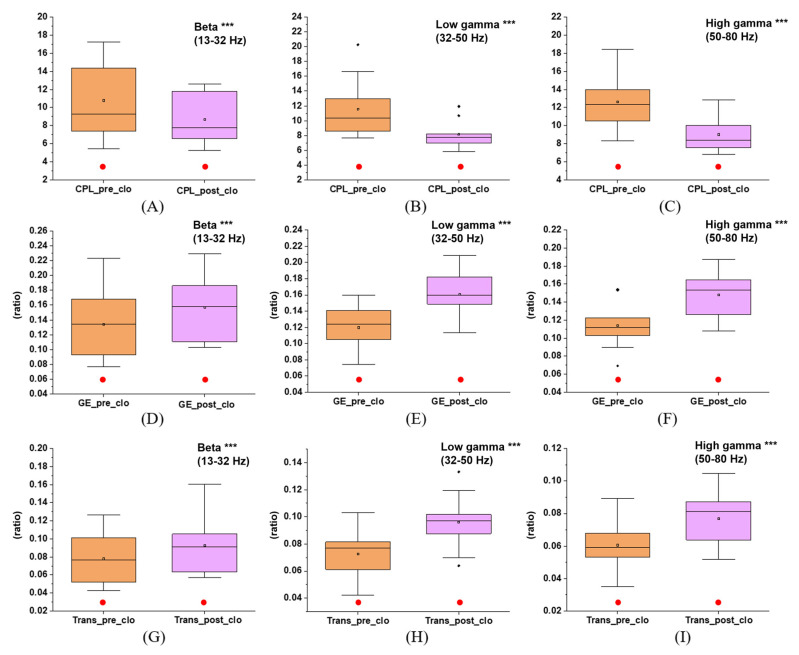
CPL, GE, and transitivity variation in stages two and five at different frequency bands: (**A**) CPL in the beta band (*p* = 0.046); (**B**) CPL in the low gamma band (*p* = 0.009); (**C**) CPL in the high gamma band (*p* = 0.002); (**D**) GE in the beta band (*p* = 0.035); (**E**) GE in the low gamma band (*p* = 0.003); (**F**) GE in the high gamma band (*p* = 0.002); (**G**) Transitivity in the beta band (*p* = 0.038); (**H**) Transitivity in the low gamma band (*p* = 0.002); (**I**) Transitivity in the high gamma band (*p* = 0.007). *** indicates that the difference in the diagram is significant (*p* < 0.05) in the paired–samples *t*-test. The solid red circle indicates no gender difference for this parameter.

Furthermore, as illustrated in Figure 7 and Supplementary Table S4, the comparison of stages two and five also showed better results in the post-VR section: in the beta band, CPL_stage4_ (decreased, *p* = 0.046), GE_stage4_ (increased, *p* = 0.035), and transitivity_stage4_ (increased, *p* = 0.038); in the low gamma band, CPL_stage4_ (decreased, p = 0.009), GE_stage4_ (increased, *p* = 0.003), and transitivity_stage4_ (increased, *p* = 0.002); and in the high gamma band, CPL_stage4_ (decreased, *p* = 0.002), GE_stage4_ (increased, *p* = 0.002), and transitivity_stage4_ (increased, *p* = 0.007). Moreover, CC_stage5_ > CC_stage2_ was significantly verified in some nodes’ low and high gamma bands (Supplementary Table S5). There were no gender differences in CPL, GE, or transitivity (Supplementary Table S6).

## 4. Discussion

In our research, we propose that immersive VR activates specific functional connectivity and improves the performance of small-world networks, i.e., the integration and segregation of network were both improved. Furthermore, such improvements are not transient and can be maintained for a period after the VR intervention ends.

Makarov et al. discovered that visual information processing and decision-making in cognitive tasks in the form of realistic two-dimensional tables increased BC values and dynamically bound the distributed functional network to shape a more uniform distribution in the gamma band [31]. Consistently, our study further demonstrated that visual stimuli and decision-making in the three-dimensional VR game would also trigger dynamically significant changes in functional networks, including quantitative metrics, information flows, and hubs, all of which are concentrated in high-frequency bands.

MI analysis was used to quantify the nonlinear amplitude interdependence between channels to attain the metrics and pathways covering network performance. We plotted the difference in the MI matrix between the VR and pre-VR stages and observed apparent interactive enhancements in the four blocks presented in Figure 3. Thereinto, the stronger correlation of the posterior region (block ①) could be divided into three cases: (1) enhancements within the primary visual area (V1) of the occipital lobe (MI values between O1 and O2 increased), (2) enhancements between the occipital and posterior temporal lobes, and (3) enhancements between the occipital and posterior parietal lobes. In case (1), colored objects in a full black-and-white virtual environment strongly activated the V1, an “early” visual hierarchy to process minor visual stimuli such as color changes. Considering cases (2) and (3), the interactive increase in the occipitotemporal and occipitoparietal areas conformed to the ventral and dorsal flows of the two-stream hypothesis, which is a neural model of visual response. Visual information travels through two pathways after leaving the occipital lobe: the ventral flow associated with object recognition enters the temporal lobe, and the dorsal flow related to spatial localization terminates in the parietal lobe. Thus, ventral and dorsal flows, suggested in the ‘late’ hierarchy, are referred to as ‘what pathway’ and ‘where pathway,’ respectively [40]. A growing body of literature is devoted to validating this model by measuring neuroimaging data of participants who watched two-dimensional media and then locating the visual signal source in the occipitotemporal and occipitoparietal areas [41,42,43]. In this study, we discovered both flows in response to three-dimensional VR intervention rather than realistic two-dimensional media, whose function met the process by which participants recognized and located objects in the VR puzzles. Furthermore, the strengthened occipitoparietal connections in the alpha-beta bands were more prominent than those in the low–high gamma bands, suggesting that the dorsal flow was mainly in the pattern of alpha–beta oscillations to top–down control the ventral stream and V1 [42]. The strengthened interactions in the frontal lobe (block ②) and between the frontal and posterior areas (blocks ③–④) implied that VR could activate the advanced cognition of the frontal lobe, and make the frontal and posterior regions cooperated more closely to converge visual information into cognitive loads. These changes could be sustained because of the enhancements 20 min after the VR game. In contrast, the improved functional connectivity within the posterior area was much weaker than the interactions involving the frontal lobe, indicating more extended maintenance of the activated cognition than the visual response. Nevertheless, the increased interaction within the frontal lobe after VR was more pronounced than during the VR game. This contradicts our conjecture that participants who solve puzzles should have the most activated cognition. There might be a ‘lay-back’ phenomenon of cognitive activation that has not been studied. We suspect that participants may experience mild cybersickness when initially playing VR, which somewhat inhibits cognitive activation during VR and causes an abnormal delay. This issue should be addressed in future studies.

We graphically represented the hubs and the most distinct functional connections among hubs by setting thresholds to investigate the neuroplasticity of how the small-world organization economically tuned information flows under VR intervention. Hubs play a vital role in assembling a specific functional cluster and sufficiently conveying information among clusters [32]. Hubs and robust pathways are highly relevant for a dynamic organization in functional networks [44]. As illustrated in Figure 4, the strengthened pathways between hubs in the low and high gamma bands indicate that VR could lead to a small-world network with greater robustness and broader distribution. Furthermore, the VR intervention in the low gamma band triggered paths between the occipital and prefrontal areas, and induced a sophisticated sub-network between the prefrontal and motor neural regions in the frontal lobe. This synergism reflects the dynamic allocation of attentional resources from the occipital lobe’s visual response to the prefrontal lobe’s decision-making and the motion control of the neural motor regions, thereby completing VR tasks. In the high gamma band, the robust functional pathways between the occipital, temporal, and ventrolateral prefrontal regions suggested strongly synchronized oscillations between these nodes. It further provides evidence for the structure of the visual ventral flow based on the occipital and temporal areas, with a possible expansion into the ventrolateral prefrontal nodes under comprehensive three-dimensional stimuli [45]. Moreover, the small-world network was more significant in the posterior visual functional sub-network during the VR intervention than in the pre-VR resting state, demonstrating that the visual neurophysiological events were mainly in the high gamma band [46]. However, the correlations between hubs were not strengthened in the beta band. The spontaneous functional oscillations in the beta rhythms were reported to be extremely stable when observing the visual scene of ecological nature. Conversely, the synthetic stimulation would disturb the functional connectivity in the beta band, which might be the reason for the decreased interactions between hubs [47]. The reason for the decreased functional connectivity among hubs, and increased integration and segregation in the beta band should be further explored in future studies. In this study, we set the threshold of 30% to detect hubs and found that these nine putative hubs could capture the VR-elicited neural informatic pattern. The thresholds setup should be determined based on the experiment setup to identify the specific neural interaction pattern [48]. Additionally, it was observed that there would always be a channel in the occipital lobe (O1 or O2) with a significant increase in the low and high gamma bands, indicating strong lateralization. We assumed that this prominent phenomenon was related to ocular dominance and the primary visual processing region, indicating that the channel promoted in the gamma band should be aligned with this participant’s main visual processing area.

Empirical research has attested that a few long connections are scattered among excessively short connections in the cerebral networks [49]. Brain regions might possibly interact functionally with their adjacent areas because of the lower metabolic cost of connections among anatomically neighboring cortex regions. However, EEG signal is especially difficult to estimate the correlation between adjacent cortices, because several extracranial electrodes of a nearby area may simultaneously catch the signal of a single source and falsely conclude a high interaction between these electrode nodes. In contrast, certain functional connections among spatially distant cortices are required to speed up data transmission [50,51]. Functional networks are natural fits for small-world networks, whose segregation and integration can reflect distributed anatomical modules and the efficiency of modulating pathways among distributed regions, respectively. Accordingly, the stronger the features of a small-world network, the better its capacity for information-processing and -transmission [52]. In our study, global integration, global segregation, and local segregation of functional networks in all individual frequency bands were measured using indicators from the brain connectivity toolbox, and improved significantly in high-frequency bands during and even after VR. We initially hypothesized that detailed changes in integration and segregation would vary across frequency bands with specific rhythmic characteristics. However, the results of CPL, GE, and transitivity were similar in all frequency bands. Specifically, the CPL decreased significantly while the GE increased in the high-frequency bands during the VR intervention. The improved global integration in the functional network implies that networks would propagate electrophysiological signals more efficiently, indicating that energy and substance consumption would be more economical under three-dimensional loads. The CPL was lesser in the post-VR resting state than in pre-VR while the GE increased in the high-frequency bands. It is possible that the VR-increased global integration of functional networks could be sustained for some time. There was also a significant overall increase in CC in the high-frequency bands during the VR game and in the post-VR stage compared to the pre-VR stage. This improved local segregation indicates that the individual nodes bind to form a denser functional clique for specialized information-processing under three-dimensional stimuli [53]. The plentiful functional clusters induced by VR remained after VR intervention, implying the possibility of structural module formation. The significant improvement in transitivity during the VR game and in the post-VR stage compared with the pre-VR stage demonstrated that VR could elicit greater global segregation. This reveals that the hierarchy and diversity of functional differentiation mechanisms in the network were initially facilitated by VR intervention and further sustained after VR. Moreover, the comparisons between stages two and five revealed that VR could elicit an aftereffect on the spontaneous and endogenous neural signals [24,25].

Clinical evidence suggests that psychiatric and neurological disorders, such as schizophrenia, depression, Parkinson’s disease, and Alzheimer’s disease, have disrupted functional networks [54,55,56]. Therefore, we speculate that the VR-induced neural functional plasticity inferred by neurological bases will also be synchronized in these disorders, emerging with great potential to include VR within neurotherapeutics. Using this hypothesis, we are conducting further studies to explore the therapeutic effects of long-term VR intervention in patients with Alzheimer’s disease by evaluating small-world organizations in different intervention phases.

### Limitations

First, the functional network was represented by EEG signal analysis at the electrode level, reflecting practical cortical networks with good time resolution. However, the field spread of neuronal signals caused a ‘blurring effect’, namely volume conduction, with a single source in the cortex being captured by multiple electrodes [57]. We used bin-based MI analysis to detect the VR-induced nonstationary functional coupling, but MI was particularly susceptible to volume conduction effects [58,59]. In addition, the neural pathways between the neighboring hubs might be invalid because the distance of electrodes was closer than 5 cm (Figure 4), and threshold analysis may dismiss more profound neural activity. This could result in bias in network evaluation. In future studies, we will adopt the weighted phase index or complex Pearson correlation coefficient to analyze EEG data, and further test VR-induced signals using functional magnetic resonance imaging with high spatial resolution to validate alterations in small-world networks [60,61,62]. Second, the gender imbalance in the experimental setup was due to the limited number of participants. Although we observed no significant difference between male and female network parameters; the partial eta squares (η^2^) in the one-way repeated measures ANOVA for CPL, GE, and transitivity all above 0.14 also indicated a large effect size for samples [63]. Convening larger groups of participants with more balanced sex still should be considered in future studies. Third, we excluded the FP1 and FP2 electrodes that covered parts of the prefrontal cortex because of poor signal quality. The remaining ten frontal electrodes (F7, F3, F4, F9, FT9, FC5, FC1, FC2, FC6, and FT10) accounting for one-third of the total EEG electrodes could represent the robust performance of the frontal lobe. Nevertheless, in future studies, we should utilize another VR setup with a smaller eyeshade to avoid EEG noise in these two channels caused by headset pressure. Finally, the observed neuroplasticity in this study could be induced by the giddiness in the novel 3D environment but not the VR stimulation itself. Although subjects experienced pilot test levels 1–5 of the VR game, which could make them acclimate to the 3D environment to some extent. Further study should still be carried out to perform the same experiments on consecutive days, by which we could evaluate whether the neuroplasticity induced by VR decreases with increasing days and may completely exclude the interference of cybersickness.

## 5. Conclusions

In this study, MI analysis and the most robust pathways among hubs provided evidence for the visual two-stream hypothesis from the perspective of three-dimensional visual stimulation. All the results reflect that VR can lead to a widely distributed and highly hierarchical small-world organization in high-frequency bands. The network has numerous function-specialized cliquishness for particular responses and many long pathways to transmit information efficiently. Notably, such a small-world network with enhanced information processing and exchanging capabilities can remain 20–30 min after the VR game. Our results could help to understand better the specific neuroplasticity of how small-world networks dynamically reorganize, and improve their integration and segregation in response to comprehensive three-dimensional stimulation.

## Data Availability

The data presented in this study are available on request from the corresponding author. The data are not publicly available due to institutional review board restrictions.

## Supplementary Materials

The following supporting information can be downloaded at https://www.mdpi.com/article/10.3390/brainsci12121630/s1, Figure S1: Difference in mutual information between during VR intervention and pre-VR in open eyes in the alpha band; Figure S2: Difference in mutual information between post-VR in open eyes and pre-VR in open eyes in the alpha band; Figure S3: Distribution of the top 30% correlation values of the difference in mutual information between during VR intervention and pre-VR in open eyes; Figure S4: Difference in mutual information between post-VR in closed eyes and pre-VR in closed eyes in the alpha, beta, low gamma, and high gamma bands; Figure S5: The mutual information among 9 hubs in VR intervention and pre-VR in open-eyes in the beta, low gamma, and high gamma bands; Table S1: The values of graph measures—betweenness centrality values of a single lateralized channel in the occipital lobe (O1 or O2) which have a significant improvement between during VR and pre-VR in open-eyes for ten subjects; *p*-values ^a^ of the paired–samples *t*-test; *p*-values ^b^ of the Mann–Whitney U test *p*-values; Table S2: The values of graph measures—characteristic path length, global efficiency, and transitivity—pre-VR open eyes, during VR, and post-VR open eyes in the beta, low gamma, and high gamma bands; *p*-values ^a^ of One-way repeated–measures ANOVA; *p*-values ^b^ of post-hoc *t*-test; Table S3: The values of graph measures—clustering coefficiency—pre-VR in open-eyes, during VR, and post-VR in open-eyes, in the beta, low gamma, and high gamma bands; the *p*-values ^a^ of One-way repeated measures ANOVA; the *p*-values ^b^ of post-hoc *t*-test; Table S4: The values of graph measures—characteristic path length, global efficiency, and transitivity—pre- and post-VR in close-eyes; *p*-values of paired–samples *t*-test; Table S5: The values of graph measures—clustering coefficiency—pre- and post-VR in close-eyes, in the low gamma and high gamma bands; *p*-values of paired–samples *t*-test; Table S6: The values and results of Mann–Whitney U test of global graph measures—charactheristic path length, global efficiency, and transitivity—pre-VR in open-eyes, pre-VR in close-eyes, during VR, post-VR in open-eyes, and post-VR in close-eyes, in the beta, low gamma, and high gamma bands.
