# Supplementary material for: Evaluating the Alterations Induced by Virtual Reality in Cerebral Small-World Networks Using Graph Theory Analysis with Electroencephalography
Source: Brain Sci. 2022 Nov 28;12(12):1630. doi: 10.3390/brainsci12121630 (PMC9776076; doi:10.3390/brainsci12121630)
Supplement: Supplementary file 1 [file brainsci-12-01630-s001.zip › brainsci-2025656-supplementary.pdf]

Supplementary Figure S1

Difference in mutual information between during VR intervention and pre–VR in open eyes in the alpha band.

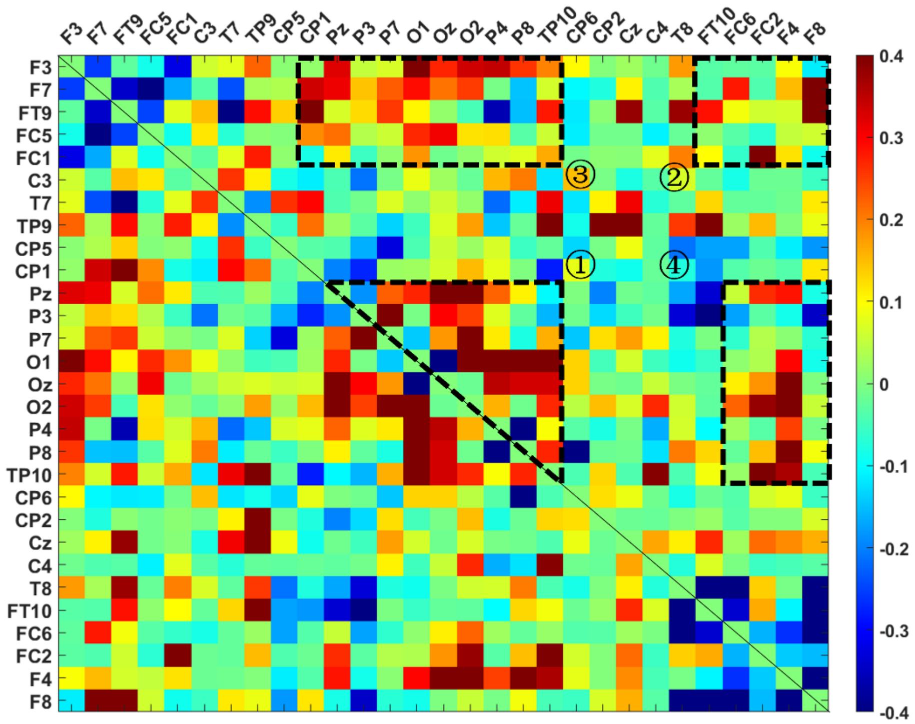

We calculated and summarized the difference of mutual information (MI) among 29 channels in the alpha band for ten participants between during VR intervention and pre–VR in open–eyes. Below consequences being similar with the beta, low gamma, and high gamma bands could be observed:

Because of the symmetry of the mutual information matrix, we only marked blocks ①–④ in the half plot above the diagonal to illustrate the results more briefly and clearly. There was an improvement of MI in four blocks, as illustrated in blocks ①–④, which was corresponding to the communications within the posterior area and frontal lobe, and in the posterior–to–anterior pattern.

Supplementary Figure S2

Difference in mutual information between post–VR in open eyes and pre–VR in open eyes in the alpha band.

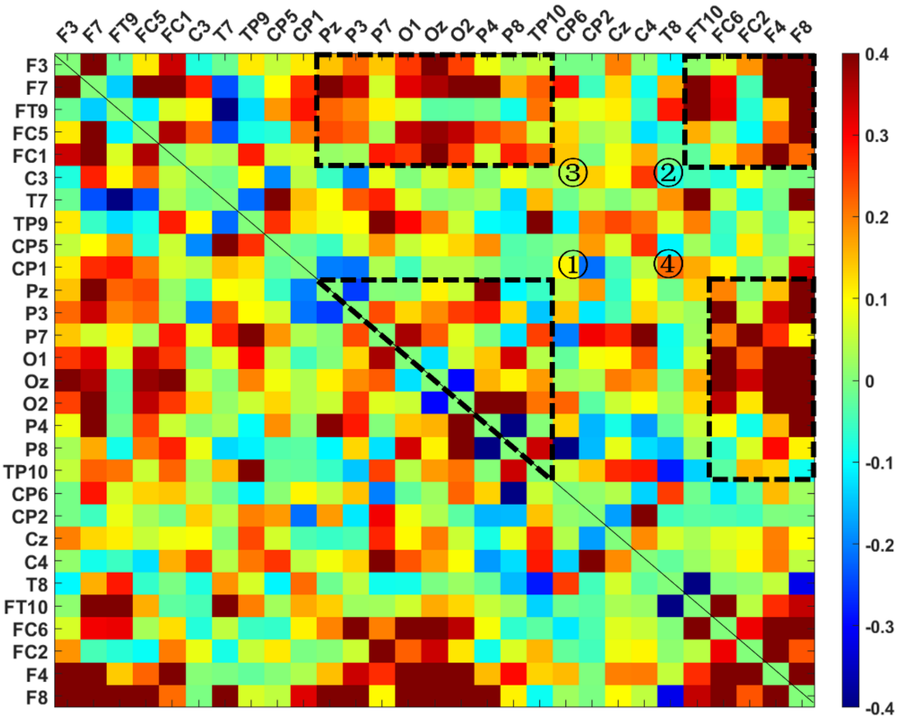

We calculated and summarized the difference of mutual information (MI) among 29 channels in the alpha band for ten participants between post–VR in open–eyes and pre–VR in open–eyes. Below consequences being similar with the beta, low gamma, and high gamma bands could be observed:

Because of the symmetry of the mutual information matrix, we only marked blocks ①–④ in the half plot above the diagonal to illustrate the results more briefly and clearly. There was an improvement of MI in four blocks, as illustrated in blocks ①–④, which was corresponding to the communications within the posterior area and frontal lobe, and in the posterior–to–anterior pattern.

Supplementary Figure S3

Distribution of the top 30% correlation values of the difference in mutual information between during VR intervention and pre–VR in open–eyes in the beta band as (A), and between post–VR in open–eyes and pre–VR in open–eyes in the beta band as (B).

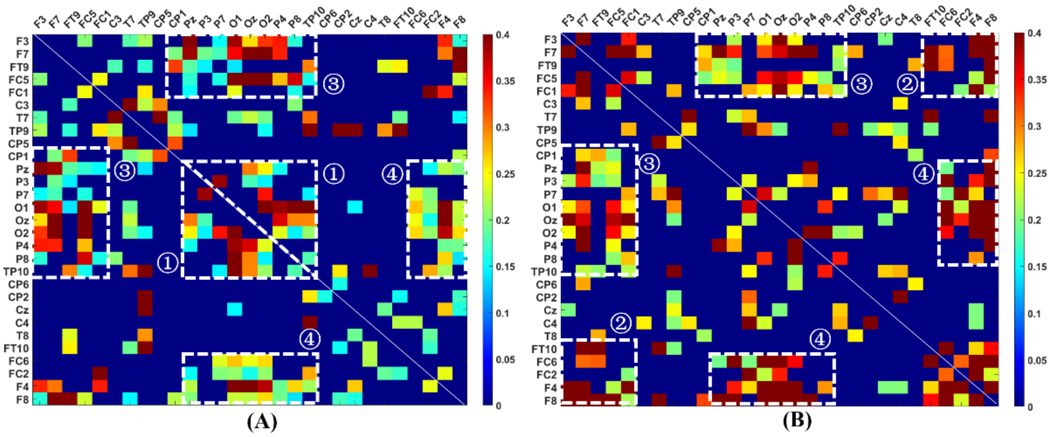

(A) (B)

We reserved the top 30% correlation values to observe their distributions.

We found that there were several regions larger than 24 grids, where more than half of correlation corresponded to these top values.

Specifically,

Block ① in (A) contained 36 grids, 19 of which corresponded to the top 30% values (ratio: 52.8%);

Block ② in (B) contained 25 grids, 14 of which corresponded to the top 30% values (ratio: 56%);

Block ③ in (A) contained 50 grids, 31 of which corresponded to the top 30% values (ratio: 62%);

Block ③ in (B) contained 50 grids, 30 of which corresponded to the top 30% values (ratio: 60%);

Block ④ in (A) contained 36 grids, 23 of which corresponded to the top 30% values (ratio: 63.9%);

Block ④ in (B) contained 36 grids, 21 of which corresponded to the top 30% values (ratio: 58.3%).

We combined these blocks ①–④ to label them in all frequency bands in other difference of MI figures. Because of the symmetry of the mutual information matrix, we only marked these blocks in the half plot above the diagonal to illustrate the results more briefly and clearly.

Supplementary Figure S4

Difference in mutual information between post–VR in closed eyes and pre–VR in closed eyes in the alpha, beta, low gamma, and high gamma bands.

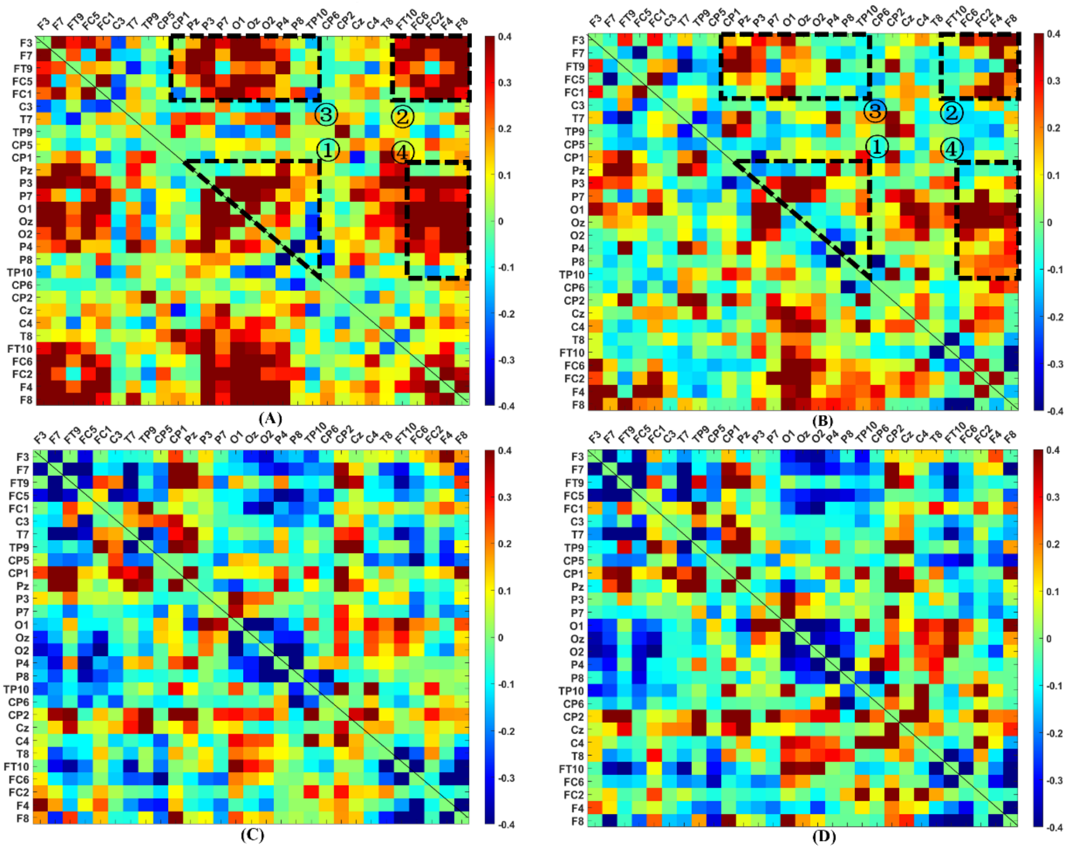

We calculated and summarized the difference of mutual information (MI) among 29 channels in six frequency bands for ten participants between post–VR and pre–VR in close–eyes. It has been proved that VR intervention was more likely to have an effect in high–frequency bands, thus we only displayed values in alpha, beta, low gamma, and high gamma bands. Below consequences could be observed:

Because of the symmetry of the mutual information matrix, we only marked blocks ①–④ in the half plot above the diagonal to illustrate the results more briefly and clearly. There was an improvement of MI in the alpha and beta bands, as illustrated in block ①–④ in figure (A) and figure (B), which was corresponding to the communications within the posterior area and frontal lobe, and in the posterior–to–anterior pattern.

In low gamma and high gamma bands, the interactions within frontal and occipital lobes were no longer strengthened a lot or even weaker, and the connections in the posterior–to–anterior pattern became shorter since it could only be observed the enhancement of communications from frontal and occipital lobes to the central sub–network.

Supplementary Figure S5

The mutual information among 9 hubs in VR intervention and pre–VR in open–eyes in the beta, low gamma, and high gamma bands.

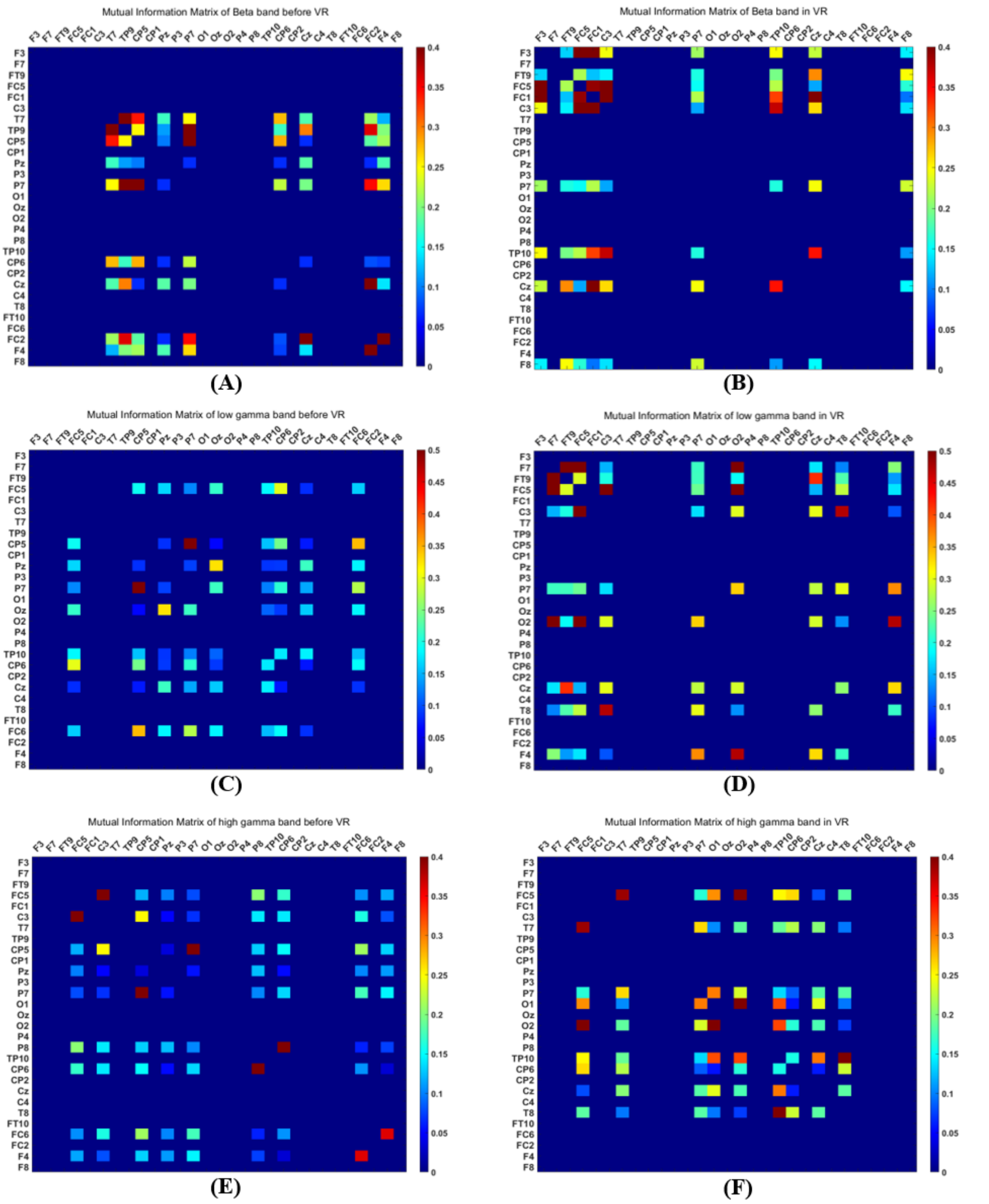

We calculated and summarized the mutual information (MI) among 9 hubs in six frequency bands for ten participants in VR intervention and pre–VR in open–eyes. It has been proved that VR intervention was more likely to have an effect in high–frequency bands, thus we only displayed values in the beta, low gamma, and high gamma bands. Below consequences could be observed:

There was an improvement of MI in the low gamma and high gamma bands, but we couldn't observe this phenomenon in the beta band.

Supplementary Table S1

The values of graph measures – betweenness centrality values of a single lateralized channel in the occipital lobe (O1 or O2) which have a significant improvement between during VR and pre–VR in open–eyes for ten subjects; p–values[a] of the paired–samples t–test; p–values[b] of the Mann–Whitney U test p–values.

		Beta		Low Gamma		High Gamma	
No.	Channel	Pre–VR Open Eyes	Post–VR Open Eyes	Pre–VR Open Eyes	Post–VR Open Eyes	Pre–VR Open Eyes	Post–VR Open Eyes
1	O1	8	2	2	6	44	56
2	O1	24	0	6	10	0	14
3	O1	20	48	14	52	8	56
4	O1	28	0	4	4	4	18
5	O1	2	10	8	4	2	2
6	O2	0	14	0	26	0	8
7	O2	28	18	8	42	0	4
8	O2	12	32	34	60	16	42
9	O2	10	68	24	42	10	70
10	O2	8	34	2	62	12	34
Mean		14	22.6	10.2	30.8	9.6	30.4
p–value[a]		0.3287		0.0105 (***)		0.0078 (***)	
p–value[b]		0.4222	0.1702	0.0513	0.2516	0.8175	0.6475

p–value[a] was calculated using paired–samples t–test;

p–value[b] was calculated using the Mann–Whitney U test;

*** indicates the difference is significant ($p < 0.05$) in the paired–samples t–test p–values.

Supplementary Table S2

The values of graph measures – characteristic path length, global efficiency, and transitivity – pre–VR open eyes, during VR, and post–VR open eyes in the beta, low gamma, and high gamma bands; p–values[a] of One–way repeated–measures ANOVA; p–values[b] of post–hoc t–test.

Parameter	Frequency Band	Category	Mean	*p*–Value[a]	Post–Hoc Groups	*p*–Value[b]
CPL	Beta	Pre	10.95509	<0.001***	Pre–VR	0.002*
		VR	6.76093		VR–post	0.003*
		Post	7.83317		Pre–post	0.01*
	Low gamma	Pre	12.49140	<0.001***	Pre–VR	<0.001*
		VR	6.82550		VR–post	0.002*
		Post	8.53792		Pre–post	0.003*
	High gamma	Pre	14.74589	<0.001***	Pre–VR	<0.001*
		VR	7.21147		VR–post	<0.001*
		Post	9.83538		Pre–post	0.001*
GE	Beta	Pre	0.13431	<0.001***	Pre–VR	<0.001*
		VR	0.19510		VR–post	0.001*
		Post	0.16706		Pre–post	0.003*
	Low gamma	Pre	0.11217	<0.001***	Pre–VR	<0.001*
		VR	0.19119		VR–post	<0.001*
		Post	0.15388		Pre–post	<0.001*
	High gamma	Pre	0.09672	<0.001***	Pre–VR	<0.001*
		VR	0.18065		VR–post	<0.001*
		Post	0.13839		Pre–post	<0.001*
Trans	Beta	Pre	0.07800	<0.001***	Pre–VR	<0.001*
		VR	0.11717		VR–post	0.001*
		Post	0.09778		Pre–post	0.002*
	Low gamma	Pre	0.06657	<0.001***	Pre–VR	<0.001*
		VR	0.11407		VR–post	<0.001*
		Post	0.08986		Pre–post	<0.001*

	High gamma	Pre	0.04863	<0.001***	Pre–VR	<0.001*
		VR	0.09464		VR–post	<0.001*
		Post	0.07103		Pre–post	0.001*

p–values[a] was calculated using One–way repeated–measures ANOVA;
p–values[b] was calculated using post–hoc t–test;
*** indicates the difference is significant (p < 0.05) in One–way repeated measures ANOVA;
* indicates the difference is significant (p < 0.16) in post–hoc t–test after Bonferroni conrrection.

We calculated and summarized the average values of CPL, GE, and transitivity in the beta, low gamma, and high gamma frequency bands for ten participants pre–VR in open–eyes, during VR, and post–VR in open–eyes. We did the One–way repeated measures ANOVA to anlyze whether there is a difference between these three groups. And then post–hoc t–test was further performed to find the significant difference between the sub–groups, that is, pre– and during VR, during and post–VR, and pre– and post–VR. Below consequences could be observed:

For CPL, the values pre–VR were greater than they post–VR and during VR, and the values post–VR were greater than they during VR in the beta, low gamma, and high gamma bands, with a significant difference statistically verified by p–values of One–way repeated measures ANOVA (< 0.05) and p–values of post–hoc t–test (<0.016, Bonferroni corrected). CPL is the network parameter to evaluate the level of global integration.

For GE and transitivity, the values pre–VR were smaller than they post–VR and during VR, and the values post–VR were smaller than they during VR in the beta, low gamma, and high gamma bands, with a significant difference statistically verified by p–values of one–way repeated measures ANOVA (< 0.05) and p–values of post–hoc t–test (<0.016, Bonferroni corrected). GE is the network parameter to evaluate the level of global integration, and transitivity is the network parameter to evaluate the level of global segregation.

In conclusion, global integration and segregation in functional networks was improved a lot in the beta, low gamma, and high gamma bands during VR intervention.

Supplementary Table S3

The values of graph measures – clustering coefficiency – pre–VR in open–eyes, during VR, and post–VR in open–eyes, in the beta, low gamma, and high gamma bands; the p–values[a] of One–way repeated measures ANOVA; the p–values[b] of post–hoc t–test.

Channel	Frequency Band	Category	Mean	*p*–Value[a]	Post–Hoc Groups	*p*–Value[b]
F3	Beta	Pre	0.079	<.001***	Pre–VR	<0.001*
		VR	0.129		VR–post	<0.001*
		Post	0.101		Pre–post	0.002*
	Low gamma	Pre	0.068	<0.001***	Pre–VR	<0.001*
		VR	0.126		VR–post	<0.001*
		Post	0.089		Pre–post	0.006*
	High gamma	Pre	0.049	<0.001***	Pre–VR	<0.001*
		VR	0.108		VR–post	0.001*
		Post	0.071		Pre–post	0.02
F7	Beta	Pre	0.070	<0.001***	Pre–VR	<0.001*
		VR	0.119		VR–post	0.092
		Post	0.100		Pre–post	0.001*
	Low gamma	Pre	0.065	<0.001***	Pre–VR	<0.001*
		VR	0.117		VR–post	0.006*
		Post	0.097		Pre–post	<0.001*
	High gamma	Pre	0.050	<0.001***	Pre–VR	<0.001*
		VR	0.101		VR–post	0.002*
		Post	0.085		Pre–post	0.004*
FT9	Beta	Pre	0.074	<0.001***	Pre–VR	<0.001*
		VR	0.112		VR–post	0.002*
		Post	0.091		Pre–post	0.007*
	Low gamma	Pre	0.066	<0.001***	Pre–VR	<0.001*

		VR	0.114		VR–post	0.002*
		Post	0.095		Pre–post	<0.001*
	High gamma	Pre	0.050	<0.001***	Pre–VR	<0.001*
		VR	0.095		VR–post	0.003*
		Post	0.077		Pre–post	0.002*
FC5	Beta	Pre	0.077	<0.001***	Pre–VR	<0.001*
		VR	0.125		VR–post	0.006*
		Post	0.098		Pre–post	0.015*
	Low gamma	Pre	0.067	<0.001***	Pre–VR	<0.001*
		VR	0.121		VR–post	<0.001*
		Post	0.090		Pre–post	0.003*
	High gamma	Pre	0.050	<0.001***	Pre–VR	<0.001*
		VR	0.100		VR–post	0.002*
		Post	0.071		Pre–post	0.012*
FC1	Beta	Pre	0.082	<0.001***	Pre–VR	0.002*
		VR	0.126		VR–post	0.039
		Post	0.108		Pre–post	0.003*
	Low gamma	Pre	0.069	<0.001***	Pre–VR	<0.001*
		VR	0.120		VR–post	0.006*
		Post	0.093		Pre–post	<0.001*
	High gamma	Pre	0.047	<0.001***	Pre–VR	<0.001*
		VR	0.097		VR–post	<0.001*
		Post	0.068		Pre–post	0.002*
C3	Beta	Pre	0.081	0.001***	Pre–VR	0.002*
		VR	0.116		VR–post	0.001*
		Post	0.094		Pre–post	0.407

	Low gamma	Pre	0.068	<0.001***	Pre–VR	0.007*
		VR	0.110		VR–post	<0.001*
		Post	0.085		Pre–post	0.028
	High gamma	Pre	0.047	<0.001***	Pre–VR	<0.001*
		VR	0.088		VR–post	0.001*
		Post	0.061		Pre–post	0.029
T7	Beta	Pre	0.078	<0.001***	Pre–VR	<0.001*
		VR	0.119		VR–post	0.008*
		Post	0.091		Pre–post	0.013*
	Low gamma	Pre	0.070	<0.001***	Pre–VR	<0.001*
		VR	0.118		VR–post	0.003*
		Post	0.088		Pre–post	0.005*
	High gamma	Pre	0.055	<0.001***	Pre–VR	<0.001*
		VR	0.097		VR–post	0.003*
		Post	0.070		Pre–post	0.009*
TP9	Beta	Pre	0.077	0.001***	Pre–VR	0.005*
		VR	0.119		VR–post	0.009*
		Post	0.092		Pre–post	0.011*
	Low gamma	Pre	0.066	<0.001***	Pre–VR	0.002*
		VR	0.119		VR–post	0.005*
		Post	0.086		Pre–post	0.01*
	High gamma	Pre	0.049	<0.001***	Pre–VR	<0.001*
		VR	0.099		VR–post	0.003*
		Post	0.067		Pre–post	0.002*
CP5	Beta	Pre	0.078	0.004***	Pre–VR	<0.001*
		VR	0.102		VR–post	0.241

		Post	0.093		Pre–post	0.028
	Low gamma	Pre	0.065	0.001***	Pre–VR	0.002*
		VR	0.104		VR–post	0.003*
		Post	0.084		Pre–post	0.017*
	High gamma	Pre	0.047	0.001***	Pre–VR	0.001*
		VR	0.084		VR–post	0.003*
		Post	0.064		Pre–post	0.002*
CP1	Beta	Pre	0.075	<0.001***	Pre–VR	<0.001*
		VR	0.111		VR–post	0.003*
		Post	0.092		Pre–post	0.009*
	Low gamma	Pre	0.061	<0.001***	Pre–VR	<0.001*
		VR	0.108		VR–post	0.009*
		Post	0.088		Pre–post	<0.001*
	High gamma	Pre	0.045	<0.001***	Pre–VR	<0.001*
		VR	0.092		VR–post	0.016
		Post	0.071		Pre–post	0.002
Pz	Beta	Pre	0.076	<0.001***	Pre–VR	<0.001*
		VR	0.115		VR–post	0.014
		Post	0.093		Pre–post	0.008
	Low gamma	Pre	0.067	<0.001***	Pre–VR	<0.001*
		VR	0.108		VR–post	0.007
		Post	0.091		Pre–post	0.009
	High gamma	Pre	0.049	0.001***	Pre–VR	<0.001*
		VR	0.094		VR–post	0.001
		Post	0.075		Pre–post	0.002
P3	Beta	Pre	0.081	0.004***	Pre–VR	0.007

		VR	0.107		VR–post	0.001*
		Post	0.101		Pre–post	0.005*
	Low gamma	Pre	0.068	<0.001***	Pre–VR	<0.001*
		VR	0.104		VR–post	0.001*
		Post	0.093		Pre–post	0.003*
	High gamma	Pre	0.049	0.001***	Pre–VR	<0.001*
		VR	0.088		VR–post	0.002*
		Post	0.076		Pre–post	0.001*
P7	Beta	Pre	0.078	<0.001***	Pre–VR	<0.001*
		VR	0.116		VR–post	0.014*
		Post	0.101		Pre–post	0.006*
	Low gamma	Pre	0.065	<0.001***	Pre–VR	<0.001*
		VR	0.114		VR–post	0.012*
		Post	0.090		Pre–post	0.003*
	High gamma	Pre	0.047	<0.001***	Pre–VR	0.007*
		VR	0.093		VR–post	0.002*
		Post	0.070		Pre–post	0.011*
O1	Beta	Pre	0.086	0.001***	Pre–VR	0.007*
		VR	0.134		VR–post	0.009*
		Post	0.109		Pre–post	0.006*
	Low gamma	Pre	0.074	<0.001***	Pre–VR	<0.001*
		VR	0.130		VR–post	0.023
		Post	0.098		Pre–post	0.015*
	High gamma	Pre	0.056	<0.001***	Pre–VR	<0.001*
		VR	0.107		VR–post	0.002*
		Post	0.080		Pre–post	0.015*

Oz	Beta	Pre	0.084	<0.001***	Pre–VR	0.001*
		VR	0.133		VR–post	0.005*
		Post	0.111		Pre–post	<0.001*
	Low gamma	Pre	0.072	<0.001***	Pre–VR	<0.001*
		VR	0.123		VR–post	0.011*
		Post	0.098		Pre–post	0.005*
	High gamma	Pre	0.054	0.001***	Pre–VR	0.002*
		VR	0.102		VR–post	0.005*
		Post	0.080		Pre–post	0.01*
O2	Beta	Pre	0.081	<0.001***	Pre–VR	<0.001*
		VR	0.135		VR–post	0.009*
		Post	0.108		Pre–post	<0.001*
	Low gamma	Pre	0.071	<0.001***	Pre–VR	<0.001*
		VR	0.131		VR–post	0.002*
		Post	0.095		Pre–post	0.002*
	High gamma	Pre	0.055	<0.001***	Pre–VR	0.001*
		VR	0.110		VR–post	0.012*
		Post	0.076		Pre–post	0.001*
P4	Beta	Pre	0.081	<0.001***	Pre–VR	<0.001*
		VR	0.121		VR–post	0.004*
		Post	0.100		Pre–post	<0.001*
	Low gamma	Pre	0.071	<0.001***	Pre–VR	<0.001*
		VR	0.115		VR–post	0.011*
		Post	0.091		Pre–post	0.001*
	High gamma	Pre	0.056	0.002***	Pre–VR	0.002*
		VR	0.096		VR–post	0.04

		Post	0.073		Pre–post	0.013*
		Pre	0.084		Pre–VR	0.002*
	Beta	VR	0.124	<0.001***	VR–post	0.007*
		Post	0.100		Pre–post	<0.001*
		Pre	0.070		Pre–VR	<0.001*
P8	Low gamma	VR	0.123	0.001***	VR–post	<0.001*
		Post	0.084		Pre–post	0.013*
		Pre	0.054		Pre–VR	0.017*
	High gamma	VR	0.102	<0.001***	VR–post	0.009*
		Post	0.064		Pre–post	<0.001*
		Pre	0.073		Pre–VR	0.011*
	Beta	VR	0.120	<0.001***	VR–post	0.005*
		Post	0.090		Pre–post	0.006*
		Pre	0.057		Pre–VR	<0.001*
TP10	Low gamma	VR	0.121	<0.001***	VR–post	<0.001*
		Post	0.081		Pre–post	0.002*
		Pre	0.039		Pre–VR	<0.001*
	High gamma	VR	0.103	<0.001***	VR–post	0.002*
		Post	0.061		Pre–post	<0.001*
		Pre	0.077		Pre–VR	<0.001*
	Beta	VR	0.105	<0.001***	VR–post	0.013*
		Post	0.090		Pre–post	<0.001*
CP6		Pre	0.063		Pre–VR	0.015*
	Low gamma	VR	0.100	0.001***	VR–post	0.017*
		Post	0.079		Pre–post	<0.001*
	High gamma	Pre	0.046	<0.001***	Pre–VR	<0.001*

		VR	0.079		VR–post	0.007*
		Post	0.060		Pre–post	0.001*
CP2	Beta	Pre	0.077	<0.001***	Pre–VR	0.016*
		VR	0.111		VR–post	<0.001*
		Post	0.090		Pre–post	<0.001*
	Low gamma	Pre	0.065	<0.001***	Pre–VR	0.017*
		VR	0.105		VR–post	0.27
		Post	0.087		Pre–post	0.001*
	High gamma	Pre	0.047	<0.001***	Pre–VR	<0.001*
		VR	0.089		VR–post	0.013*
		Post	0.069		Pre–post	<0.001*
Cz	Beta	Pre	0.079	<0.001***	Pre–VR	<0.001*
		VR	0.121		VR–post	0.009*
		Post	0.098		Pre–post	0.015*
	Low gamma	Pre	0.063	0.001***	Pre–VR	<0.001*
		VR	0.115		VR–post	0.003*
		Post	0.089		Pre–post	<0.001*
	High gamma	Pre	0.041	<0.001***	Pre–VR	<0.001*
		VR	0.096		VR–post	0.011*
		Post	0.065		Pre–post	0.001*
C4	Beta	Pre	0.080	0.001***	Pre–VR	0.007*
		VR	0.106		VR–post	0.013*
		Post	0.098		Pre–post	<0.001*
	Low gamma	Pre	0.080	0.001***	Pre–VR	0.01*
		VR	0.106		VR–post	0.016*
		Post	0.098		Pre–post	<0.001*

	High gamma	Pre	0.050	<0.001***	Pre–VR	<0.001*
		VR	0.080		VR–post	0.007*
		Post	0.067		Pre–post	<0.001*
T8	Beta	Pre	0.078	<0.001***	Pre–VR	<0.001*
		VR	0.109		VR–post	0.003*
		Post	0.088		Pre–post	<0.001*
	Low gamma	Pre	0.067	<0.001***	Pre–VR	0.003*
		VR	0.110		VR–post	0.001*
		Post	0.082		Pre–post	0.002*
	High gamma	Pre	0.047	<0.001***	Pre–VR	<0.001*
		VR	0.089		VR–post	0.015*
		Post	0.064		Pre–post	<0.001*
FT10	Beta	Pre	0.070	<0.001***	Pre–VR	<0.001*
		VR	0.101		VR–post	0.002*
		Post	0.089		Pre–post	<0.001*
	Low gamma	Pre	0.061	<0.001***	Pre–VR	0.002*
		VR	0.104		VR–post	0.017*
		Post	0.088		Pre–post	<0.001*
	High gamma	Pre	0.044	<0.001***	Pre–VR	<0.001*
		VR	0.087		VR–post	0.023*
		Post	0.073		Pre–post	<0.001*
FC6	Beta	Pre	0.074	<0.001***	Pre–VR	0.001*
		VR	0.107		VR–post	0.007*
		Post	0.099		Pre–post	<0.001*
	Low gamma	Pre	0.065	<0.001***	Pre–VR	0.002*
		VR	0.106		VR–post	0.013*

		Post	0.090		Pre–post	<0.001*
	High gamma	Pre	0.047	0.001***	Pre–VR	<0.001*
		VR	0.088		VR–post	0.017*
		Post	0.073		Pre–post	<0.001*
FC2	Beta	Pre	0.085	0.001***	Pre–VR	<0.001*
		VR	0.125		VR–post	<0.001*
		Post	0.103		Pre–post	<0.001*
	Low gamma	Pre	0.070	<.001***	Pre–VR	0.007*
		VR	0.119		VR–post	0.033
		Post	0.093		Pre–post	0.005*
	High gamma	Pre	0.047	<0.001***	Pre–VR	0.003*
		VR	0.094		VR–post	0.01*
		Post	0.066		Pre–post	<0.001*
F4	Beta	Pre	0.078	<0.001***	Pre–VR	<0.001*
		VR	0.120		VR–post	<0.001*
		Post	0.104		Pre–post	<0.001*
	Low gamma	Pre	0.067	<0.001***	Pre–VR	0.016*
		VR	0.115		VR–post	<0.001*
		Post	0.098		Pre–post	<0.001*
	High gamma	Pre	0.046	0.004***	Pre–VR	<0.001*
		VR	0.096		VR–post	0.007*
		Post	0.078		Pre–post	0.003*
F8	Beta	Pre	0.067	<0.001***	Pre–VR	0.007*
		VR	0.107		VR–post	0.016*
		Post	0.102		Pre–post	0.012*
	Low gamma	Pre	0.061	<0.001***	Pre–VR	<0.001*

		VR	0.108		VR–post	0.002*
		Post	0.096		Pre–post	<0.001*
		Pre	0.045		Pre–VR	<0.001*
	High gamma	VR	0.092	<0.001***	VR–post	0.004*
		Post	0.083		Pre–post	<0.001*

p–values[a] was calculated using One–way repeated–measures ANOVA;
p–values[b] was calculated using post–hoc t–test;
*** indicates the difference is significant ($p < 0.05$) in One–way repeated measures ANOVA.
* indicates the difference is significant ($p < 0.16$) in post–hoc t–test after Bonferroni conrrection.

We calculated and summarized the average values of clustering coefficiency (CC) among 29 channels in the high–frequency bands for ten participants pre–VR in open–eyes, during VR, and post–VR in open–eyes. Below consequences could be observed:

For CC, the values pre–VR were lower than they post–VR and during VR, and the values post–VR were lower than they during VR, in most nodes (more focused in frontal and occipital lobes) of the beta band and all nodes of the low gamma and high gamma bands, with a significant difference statistically verified by p–value of One–way repeated measures ANOVA (< 0.05) and p–value of post–hoc t–test (<0.016, Bonferroni corrected).

CC is a metric to measure the degree of local segregation of a single node in functional networks.

In conclusion, local segregation of most nodes in functional networks was improved a lot in the beta band during VR intervention, especially for the nodes in frontal and occipital lobes. And the local segregation of all nodes was also improved a lot in both low and high gamma bands during VR intervention.

Supplementary Table S4

The values of graph measures – characteristic path length, global efficiency, and transitivity – pre– and post–VR in close–eyes; p–values of paired–samples t–test.

Parameters	Frequency Band	Category	Mean	Change to Pre–VR	*p*–Value (< 0.05)
Characteristic path length	Delta	Pre	6.74791	–	0.16418
		Post	5.49944	18.50%	
	Theta	Pre	7.24163	–	0.40447
		Post	6.35321	12.27%	
	Alpha	Pre	8.10255	–	0.39842
		Post	7.02932	13.25%	
	Beta	Pre	10.77853	–	0.046 (*)
		Post	8.68984	19.38%	
	Low gamma	Pre	11.56271	–	0.009 (*)
		Post	8.13223	29.67%	
	High gamma	Pre	12.64292	–	0.002 (*)
		Post	9.00519	28.77%	
Global efficiency	Delta	Pre	0.19088	–	0.24177
		Post	0.22383	17.26%	
	Theta	Pre	0.18246	–	0.39435
		Post	0.20863	14.35%	
	Alpha	Pre	0.17408	–	0.43640
		Post	0.19931	14.50%	
	Beta	Pre	0.13376	–	0.035 (*)
		Post	0.15664	17.11%	
	Low gamma	Pre	0.11970	–	0.003 (*)
		Post	0.16094	34.46%	
	High gamma	Pre	0.11370	–	0.002 (*)
		Post	0.14791	30.09%	
Transitivity	Delta	Pre	0.17445	–	0.21660
		Post	0.20690	18.60%	
	Theta	Pre	0.15974	–	0.31427
		Post	0.18880	18.20%	

	Alpha	Pre	0.14430	–	0.41946
		Post	0.16660	15.45%	
	Beta	Pre	0.07789	–	0.038 (*)
		Post	0.09245	18.69%	
	Low gamma	Pre	0.07261	–	0.002 (*)
		Post	0.09598	32.19%	
	High gamma	Pre	0.06059	–	0.007 (*)
		Post	0.07686	26.85%	

The asterisk (*) indicates the difference is significant ($p < 0.05$).

We calculated and summarized the average values of characteristic path length (CPL), global efficiency (GE), and transitivity among 29 channels in six frequency bands for ten participants pre– and post–VR in close–eyes. Below consequences could be observed:

For CPL, the value pre–VR was higher than it post–VR in the low gamma and high gamma bands, with a significant difference statistically verified by p–value (< 0.05).

For GE, the value pre–VR was lower than it post–VR in the beta, low gamma, and high gamma bands, with a significant difference statistically verified by p–value (< 0.05).

For transitivity, the value pre–VR was lower than it post–VR in the beta, low gamma, and high gamma bands, with a significant difference statistically verified by p–value (< 0.05).

CPL and GE are metrics to evaluate the degree of integration of functional networks. The results of CPL and GE indicated that the integration improved a lot after VR intervention in the beta, low gamma, and high gamma bands.

Transitivity is a metric to evaluate the degree of segregation of functional networks. The results of transitivity displayed that the segregation improved a lot after VR intervention in the beta, low gamma, and high gamma bands.

In conclusion, integration and segregation of functional networks were both improved in the open–eyes state among high–frequency bands even after the VR game.

Supplementary Table S5

The values of graph measures – clustering coefficiency – pre– and post–VR in close–eyes, in the low gamma and high gamma bands; p–values of paired–samples t–test.

Channel	Low gamma			High gamma		
	Category	Mean	*p*–Value	Category	Mean	*p*–Value
F3	Pre	0.073	0.064	Pre	0.061	0.186
	Post	0.101		Post	0.077	
F7	Pre	0.079	0.045 (*)	Pre	0.071	0.174
	Post	0.101		Post	0.084	
FT9	Pre	0.077	0.009 (*)	Pre	0.069	0.067
	Post	0.103		Post	0.088	
FC5	Pre	0.081	0.181	Pre	0.070	0.432
	Post	0.096		Post	0.077	
FC1	Pre	0.071	0.061	Pre	0.054	0.052
	Post	0.094		Post	0.069	
C3	Pre	0.071	0.204	Pre	0.054	0.227
	Post	0.086		Post	0.064	
T7	Pre	0.080	0.078	Pre	0.068	0.200
	Post	0.104		Post	0.083	
TP9	Pre	0.070	0.023 (*)	Pre	0.057	0.048 (*)
	Post	0.087		Post	0.071	
CP5	Pre	0.077	0.124	Pre	0.062	0.221
	Post	0.096		Post	0.074	
CP1	Pre	0.059	0.002 (*)	Pre	0.047	0.048 (*)
	Post	0.091		Post	0.071	
Pz	Pre	0.065	0.004 (*)	Pre	0.057	0.097
	Post	0.094		Post	0.075	
P3	Pre	0.067	0.001 (*)	Pre	0.054	0.023 (*)
	Post	0.098		Post	0.078	
P7	Pre	0.075	0.017 (*)	Pre	0.059	0.017 (*)
	Post	0.098		Post	0.079	
O1	Pre	0.075	0.002 (*)	Pre	0.069	0.029 (*)
	Post	0.103		Post	0.086	
Oz	Pre	0.078	0.013 (*)	Pre	0.073	0.089
	Post	0.102		Post	0.087	
O2	Pre	0.076	0.058	Pre	0.070	0.239
	Post	0.095		Post	0.079	
P4	Pre	0.073	0.029 (*)	Pre	0.062	0.126

	Post	0.096		Post	0.077	
P8	Pre	0.074	0.140	Pre	0.061	0.212
	Post	0.090		Post	0.072	
TP10	Pre	0.066	0.146	Pre	0.054	0.175
	Post	0.080		Post	0.065	
CP6	Pre	0.074	0.116	Pre	0.056	0.072
	Post	0.095		Post	0.075	
CP2	Pre	0.058	0.001 (*)	Pre	0.045	0.042 (*)
	Post	0.092		Post	0.069	
Cz	Pre	0.061	0.002 (*)	Pre	0.045	0.016 (*)
	Post	0.086		Post	0.064	
C4	Pre	0.072	0.036 (*)	Pre	0.054	0.025 (*)
	Post	0.096		Post	0.076	
T8	Pre	0.072	0.091	Pre	0.059	0.080
	Post	0.094		Post	0.081	
FT10	Pre	0.076	0.113	Pre	0.068	0.256
	Post	0.095		Post	0.081	
FC6	Pre	0.080	0.179	Pre	0.068	0.244
	Post	0.099		Post	0.082	
FC2	Pre	0.071	0.020 (*)	Pre	0.051	0.028 (*)
	Post	0.098		Post	0.069	
F4	Pre	0.076	0.087	Pre	0.064	0.198
	Post	0.100		Post	0.079	
F8	Pre	0.081	0.069 (*)	Pre	0.073	0.103
	Post	0.111		Post	0.096	

The asterisk (*) indicates the difference is significant ($p < 0.05$).

We calculated and summarized the average values of clustering coefficiency (CC) among 29 channels in six frequency bands for ten participants pre–VR and post–VR in close–eyes. The p–values began to be smaller than 0.05 from the low gamma band, thus we only showed results of CC in the low gamma and high gamma bands here. Below consequences could be observed:

For CC, the values pre–VR were lower than they post–VR in some nodes of the low gamma and high gamma bands, with a significant difference statistically verified by p–value (< 0.05). And the nodes with a significant improvement in the low gamma bands were more than they in high gamma bands.

CC is a metric to measure the degree of local segregation of a single node in functional networks.

In conclusion, local segregation of some nodes in functional networks was improved in the close–eyes state among high–frequency bands after the VR game, and the nodes of the low gamma band in the close–eyes state were more likely to be influenced by VR.

Supplementary Table S6

The values and results of Mann–Whitney U test of global graph measures – charactheristic path length, global efficiency, and transitivity – pre–VR in open–eyes, pre–VR in close–eyes, during VR, post–VR in open–eyes, and post–VR in close–eyes, in the beta, low gamma, and high gamma bands.

**Parameters**	**Frequency Band**	**Category**	***p*–Value[b]**	**Parameters**	**Frequency Band**	**Category**	***p*–Value[b]**
Characteristic path length	Beta	Pre, open	0.6475	Global efficiency	Beta	Pre, open	0.5167
		Pre, close	0.3833			Pre, close	0.3833
		VR	0.8333			VR	0.8333
		Post, open	0.5167			Post, open	0.5167
		Post, close	0.8333			Post, close	0.8333
	Low gamma	Pre, open	0.2667		Low gamma	Pre, open	0.2667
		Pre, close	0.8333			Pre, close	0.8333
		VR	0.8333			VR	0.6667
		Post, open	0.6667			Post, open	0.6667
		Post, close	0.6667			Post, close	0.8333
	High gamma	Pre, open	0.1167		High gamma	Pre, open	0.1167
		Pre, close	1			Pre, close	0.8333
		VR	0.8333			VR	0.8333
		Post, open	0.8333			Post, open	1
		Post, close	1			Post, close	1
Parameters	Frequency band	Category	p–valueb				
Transitivity	Beta	Pre, open	0.3888				
		Pre, close	0.3833				
		VR	1				
		Post, open	0.6667				
		Post, close	0.8333				
	Low gamma	Pre, open	0.1833				
		Pre, close	0.8333				
		VR	0.8333				

		Post, open	0.8333	
		Post, close	1	
	High gamma	Pre, open	0.1833	
		Pre, close	1	
		VR	0.8333	
		Post, open	0.6667	
		Post, close	0.8333	

p–value[b] was calculated using the Mann–Whitney U test.
